# The impact of small changes in bar closing hours on violence. The Norwegian experience from 18 cities

**DOI:** 10.1111/j.1360-0443.2011.03643.x

**Published:** 2012-03

**Authors:** Ingeborg Rossow, Thor Norström

**Affiliations:** 1 Norwegian Institute for Alcohol and Drug ResearchOslo, Norway; 2 Swedish Institute for Social ResearchStockholm, Sweden

**Keywords:** Alcohol policy, closing hours, natural experiments, Norway, time–series analyses, violence

## Abstract

**Aims:**

To estimate the effect on violence of small changes in closing hours for on-premise alcohol sales, and to assess whether a possible effect is symmetrical.

**Design, setting, and participants:**

A quasi-experimental design drawing on data from 18 Norwegian cities that have changed (extended or restricted) the closing hours for on-premise alcohol sales. All changes were ≤ 2 hours.

**Measurements:**

Closing hours were measured in terms of the latest permitted hour of on-premise trading, ranging from 1 a.m. to 3 a.m. The outcome measure comprised police-reported assaults that occurred in the city centre between 10 p.m. and 5 a.m. at weekends. Assaults outside the city centre during the same time window should not be affected by changes in closing hours but function as a proxy for potential confounders, and was thus included as a control variable. The data spanned the period Q1 2000–Q3 2010, yielding 774 observations.

**Findings:**

Outcomes from main analyses suggested that each 1-hour extension of closing hours was associated with a statistically significant increase of 4.8 assaults (95% CI 2.60, 6.99) per 100 000 inhabitants per quarter (i.e. an increase of about 16%). Findings indicate that the effect is symmetrical. These findings were consistent across three different modelling techniques.

**Conclusion:**

In Norway, each additional 1-hour extension to the opening times of premises selling alcohol is associated with a 16% increase in violent crime.

## INTRODUCTION

The fact that alcohol consumption plays a significant role for violent behaviour [1,2] and that licensed premises are ‘hot spots’ for such behaviour [2–5], suggests that strategies to prevent heavy drinking in pubs and bars are particularly relevant for curbing violence. Violence in or around licensed premises varies significantly. It tends to occur more frequently in crowded and noisy establishments and when the overall level of intoxication of patrons is high [6,7]. While a number of prevention programmes that aim at reducing sales to intoxicated patrons and violence in bars by training bar staff may have some potential [8], in this study we will address policies to regulate the availability of on-premise drinking, more specifically in terms of regulation of closing hours.

### Effects of changing on-premise trading hours: previous research

In line with traditional economic theory on physical availability and consumption of goods, a fairly extensive literature shows that restrictions on access to alcohol are, in general, effective in curbing alcohol consumption and related harm [9,10]. Within this literature some studies have addressed the impact of restrictions on trading hours for alcohol (see [11–14] for reviews). While these studies provide empirical support for the effectiveness of a change in trading hours of 2 hours or more (see [11,12]), relatively few studies have addressed smaller changes in trading hours (i.e. changes of less than 2 hours), and the findings from these studies are inconsistent [11].

Only a few studies have assessed the possible effects of changes in trading hours on violence. Based on four recent literature reviews [11–14] and additional searches in PubMed and Google Scholar (April 2011) we identified a total of nine studies in the English language which had assessed possible effects of changes in on-premise trading hours on violence (Table 1). Six of these studies addressed extended trading hours [15–20], whereas three addressed restrictions in closing hours [21–23]. Overall, the findings from these studies are not consistent. Some studies have demonstrated associations in the expected direction, i.e. an increase in violence rates following increased trading hours and vice versa, whereas other studies have reported no association or even a decrease in violence rates with increased trading hours. We will first suggest possible explanations of how changes in trading hours may affect violence rates and then address possible methodological explanations for the inconsistent findings.

**Table 1:** Studies addressing associations between changes in trading hours for on-premise alcohol sales and violence, described by first author and publication year, study design, change in trading hours, violence measure and reported relative change in violence.

*First author, year, location*	*Study design*	*Change in trading hours*	*Violence measure*	*Relative change in violence*
Duffy, 1996 [19] England and Wales	Before–after, control region	1-hour extension opening hours	Reported violent crime	NS
Graham, 1998 [21] Edinburgh	Before–after, no comparison	Restriction of extended closing hours	Assault attendances in ER	NS
Ragnarsdottir, 2002 [15] Reykjavik	Before–after, no comparison	Extension to unrestricted closing hours	Assault attendances in ER at weekend nights	+34%
Chikritzhs, 2002 [20] Perth	Before–after, control	1-hour extension closing hours at weekends	Reported assaults in/around hotel (approx. 6% of all assaults)	+70%
Duailibi, 2007 [22] Diadema	Time–series regression	Restriction closing hours from unrestricted to 11 p.m.	Reported violent crimes:	
			Homicides	−44%
			Assaults on women	−17%
Newton, 2007 [16] London	Before–after, no comparison	Extension closing hours to unrestricted	Alcohol-related assault attendances in ER	+130%
Babb, 2007 [17] London	Before–after, no comparison	Extension closing hours to unrestricted	Night-time arrests for assaults:	
			Serious	−9%
			Less serious	−5%
Hough, 2008 [18] England and Wales	Before–after, no comparison	Extension closing hours to unrestricted	Night-time violent offences:	
			Serious	−5%
			Less serious	−3%
Kypri, 2010 [23] Newcastle	Before–after, control site	1.5–2 hours restriction closing hours + other^a^	Night-time assaults in city centre	−37%

a The change in closing hours was part of a larger intervention, which also comprised lock-out 1.5 hours prior to closing hour, as well as other interventions in the bars and pubs. ER: emergency room; NS: not significant.

In their study from Australia, Chikritzhs & Stockwell [20] reported that most of the increase in assaults in or around hotels with extended closing hours was attributed to increased alcohol sales, which could be due to an increase in the number of customers, or in the amount of alcohol consumed per customer, or both. Studies from Norway have found that in bars and pubs the general level of intoxication—and the likelihood that intoxicated patrons are served—increases by the hour at night-time [24,25]. While an increase in consumption per customer increases the risk of violent incidents [6,7], an increase in the number of customers may imply an increase in crowdedness, noise and potential provocations, which are also risk factors for violence in the night-time economy [6,26]. Another possible explanation is that extended closing hours may delay the time for visiting bars and pubs and allow for longer time to ‘pre-drink’ in private homes, in which case the customers may be more intoxicated and more likely to be involved in violence in or around bars and pubs [27]. Thus, extended closing hours may lead to an increase in violence rates for several reasons, due to increased drinking either inside the bars or during private ‘pre-drinking’, or due to other risk factors associated with an increase in the number of bar patrons, or due to combinations of these.

Several methodological aspects are of relevance for the mixed findings in previous studies. A significant problem with most of the studies on trading hours and violence is weak study design and lack of controls (see Table 1); i.e. we do not know whether observed changes in violence rates can be attributed to the change in trading hours or to other factors that may affect the violence rate. Even in quasi-experimental designs with a single intervention site and a single control site (as was the case in two studies [19,23]), it is not obvious that the observed violence in the control site is an indicator of what would have happened in the intervention site had there been no intervention [23]. Violence was assessed by different measures, which reduces comparability across the studies, but different types of changes in trading hours are probably more important. If the above-mentioned mechanisms are valid, only changes in trading hours at night-time (i.e. closing hours) are of relevance here. Indeed, among the four studies with stronger designs, three studies addressed changes in closing hours and all three found associations in the expected direction [20,^22^,23]. The fourth study addressed a small extension of opening hours and found no association between the change in trading hours and violence [19].

In line with these observations, the need for further empirical studies of the impact of changes in closing hours on alcohol consumption and related harm has been stated in the above-mentioned recent literature reviews on this topic [11–13]. In particular, the following types of studies have been requested: studies applying stronger research designs [11–13], studies that relate closing hours to levels of violence [12], studies that assess symmetry in impact of extended versus restricted closing hours [11] and studies that address the possible impact of smaller changes (<2 hours) in closing hours [11]. The latter is relevant for two reasons. Smaller changes are more often politically feasible [28], and it is theoretically important to assess whether there is a continuous relationship between availability in terms of trading hours and alcohol-related harm or some threshold effect. In the present study we will address all these issues by applying data from a series of natural experiments on changes in on-premise closing hours in Norway.

### Aims of the study

In Norway, trading hours (for both on-premise and off-premise alcohol sales) are decided at the municipality level, yet within national maximum trading hours. The national ‘normal closing hours’ for on-premise sales are 12 midnight for spirits and 1 a.m. for beer/wine, and the ‘maximum closing hours’ are 3 a.m. for all types of alcoholic beverages. Patrons are, by national law, allowed to consume alcohol 30 minutes after the closing hours for sales. The municipalities may decide to extend or restrict closing hours as long as they are within the national ‘maximum closing hours’. Over the past decade many Norwegian municipalities have changed—extended or restricted—the closing hours for on-premise sales, but the changes have been relatively minor, typically less than 2 hours.

These ‘natural experiments’ provide an opportunity to assess possible consequences of small changes in on-premise closing hours and thus add to a relatively meagre literature. The purpose of this study was twofold: (i) to assess whether small changes (≤ 2 hours) in closing hours for on-premise sales have any significance for violent crime, and (ii) if so, whether the association is symmetrical (i.e. whether the association between changes in closing hours and violence is of the same magnitude when closing hours are restricted compared to extended).

## METHODS

We collected detailed information on closing hours for on-premise alcohol sales and any changes of these by telephone interviews and e-mail correspondence with key informants and access to administrative documents in the 31 largest cities in Norway. This information comprised whether, and in that case when, any change took effect, the number, type and location of the premises that were affected by the change, the reason(s) for the changes in closing hours that had occurred and other changes in regulations concerning on-premise licences.

Closing hours refer here to the time for closing alcohol sales and were measured in terms of the latest permitted trading hour of the night, ranging from 1 a.m. to 3 a.m. A closing hour at, for instance, 1.30 a.m. was coded 1.5. In most cases the change in closing hour occurred at the very beginning of a new quarter (e.g. 1 July) and applied to all beverage types and all on-premise licences. However, in two cities extended closing hours were granted to some premises over a period before they applied to all premises, and for this period intermediate values were applied in order to reflect this gradual change. Moreover, in four cities busy periods (the summer season and the party season before Christmas) were exempted from restricted closing hours and in these cases intermediate values were applied to account for these exemptions.

The choice of violence indicators was based on the following: first, interviews with key informants in the selected cities revealed that changes in closing hours applied almost exclusively to on-premise licences in the city centres. Secondly, studies from Norway [29–31] and other countries [32] show that alcohol-related violence occurs mainly at night-time at weekends. As outcome measure we thus chose the indicator that should be most sensitive to changes in closing hours; that is, the number of assaults reported to the police that occurred in the city centre at night-time (between 10 p.m. and 5 a.m.) at weekends (Friday–Saturday and Saturday–Sunday). As a control variable we included assaults outside the city centre during the same time window. This indicator should not be affected by changes in closing hours but function as a proxy for potential confounders. Monthly data on assaults (provided by the Norwegian Police Directorate) were aggregated to quarterly time–series and converted to rates per 100 000 inhabitants.

Thirteen of the cities were excluded from the analyses for one of the following reasons: (i) lack of information about closing hours and location of violent crimes (*n* = 3); (ii) unreliable data on violent crimes (*n* = 3); (iii) changes in closing hours that also affected bars and pubs outside the city centre (*n* = 1); and (iv) no change in closing hours (*n* = 6). This left us with 18 cities for the analyses, and with the data spanning the period Q1 2000–Q3 2010, we have a total of 774 observations.

### Statistical analyses

We used pooled cross-sectional time–series analysis to estimate the effect of closing hours on assaults in the city centre. As described above, the assault rate in the city periphery was included as a control variable. An obvious source of bias in such analyses is the possible presence of unobserved city differences that are linked to the dependent and independent variables. Thus we included city dummy variables, which means that only the intercity covariation over time is explored [fixed-effects (FE) models], thus avoiding the potential bias from the intercity correlations. We used the more conservative panel corrected standard errors [33], and included panel-specific parameters for estimating residual autocorrelation (STATA version 11 was used for this analysis). To assess whether a possible effect of closing hours was symmetrical, we performed separate analyses of the set of cities that had extended (*n* = 10) or restricted (*n* = 3) the closing hours.

As sensitivity tests we analysed the data applying two other methods. The first one was city-specific time–series analyses by means of autoregressive integrated moving average (ARIMA) modelling [34]. There were 43 observations for each city. Visual inspection of the series revealed that all the assault indicators were trend-free. This is also consistent with the finding that the autocorrelations were generally low and statistically insignificant. The autocorrelation at lag 1 was statistically significant for only three cities (in the range 0.3–0.6), and no series exhibited any seasonal variation. The analyses were thus performed on the raw data, as these fulfilled the stationarity requisite of ARIMA. The noise (error) term, which includes explanatory variables not considered in the model, is allowed to have a temporal structure that is modelled and estimated in terms of autoregressive or moving average parameters. The model residuals should not differ from white noise; this was tested using the Box–Ljung Q statistics (SPSS version 17.0 was used for this analysis). The city-specific estimates were pooled into an unweighted average to obtain an overall estimate of the effect of closing hours on assaults. The standard error of the pooled estimate was calculated according to the formula (where *n* denotes the number of cities):


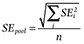


Finally, we analysed the data restricted to 2 years; that is, 1 year before and 1 year after the change in closing hours. The rationale for this method is that the influence of extraneous factors is minimized; the downside is, of course, the loss in power entailed by the few observations. In this analysis we regressed the change in city centre assaults on the change in closing hours, including change in assaults in the city periphery as control. Thirteen cities had changed closing hours once, and five cities twice, yielding 23 observations available for this analysis (SPSS version 17.0 was used for this analysis).

## RESULTS

In 10 of these cities the closing hours were restricted at one time-point, in three cities/towns the closing hours were extended, and in five cities/towns the closing hours were first extended and then restricted (see Table 2 for details). Moreover, according to our key informants the licensed premises that were affected by changes in closing hours were mainly pubs, bars and nightclubs. There are no indications that there were other changes concerning on-premise licences which were likely to have affected the outcome measure. While stated reasons for extensions of closing hours were either not given or were to serve industry interests, restrictions in closing hours were generally on the grounds that this would curb violence and public nuisance, often on the initiative of the police.

**Table 2:** Cities subject to changes in closing hours; name, inhabitants in 1000, type and extent of change to closing hours.

*City*	*Inh (')*	*Type of change*	*Extent of change in closing hours*	*Additional comments on changes*
Arendal	41	Restriction	−1.0 (3.00–2.00)	Except for summer season
Bergen	252	Both	+1.0 (2.00–3.00)	Number of premises with extended hours increased gradually before change
			−0.5 (3.00–2.30)	
Drammen	61	Extension	+0.5 (2.30–3.00)	
Fredrikstad	72	Restriction	−1.0 (2.30–1.30)	Except for summer/busy seasons
Haugesund	33	Restriction	−0.5 (1.30–1.00)	
Horten	25	Restriction	−1.0 (3.00–2.00)	
Kongsberg	24	Restriction	−1.0 (3.00–2.00)	
Kristiansand	80	Restriction	−1.0 (3.00–2.00)	
Larvik	42	Restriction	−0.5 (2.30–2.00)	
Lillehammer	26	Extension	+1.0 (2.00–3.00)	Two nightclubs had extended hours during the whole period
Molde	24	Both	+1.0 (2.00–3.00)	
			−1.0 (3.00–2.00)	
Moss	30	Restriction	−0.5 (3.00–2.30)	
Sandnes	63	Both	+1.5 (1.30–3.00)	A few nightclubs had extended hours during the whole period
			−1.5 (3.00–1.30)	
Sarpsborg	52	Restriction	−1.0 (2.30–1.30)	Except for summer/busy seasons
Stavanger	121	Both	+1.5 (1.30–3.00)	A few nightclubs had extended hours during the whole period
			−1.5 (3.00–1.30)	
Trondheim	168	Both	+1.0 (2.00–3.00)	Number of premises with extended hours increased gradually before change
			−1.0 (3.00–2.00)	
Tønsberg	39	Restriction	−1.0 (3.00–2.00)	Except for summer season
Ålesund	42	Extension	+2.0 (1.00–3.00)	

The outcome from the FE model (Table 3) suggested that each 1 hour of extension of closing hours was associated with a statistically significant increase in the number of assaults of 4.8 cases per 100 000 inhabitants per quarter [95% confidence interval (CI): 2.60–6.99]. Taking the mean number of the assault rate into account (29.2), this implies a relative increase in assaults of 16% (4.8/29.2 = 0.164) per extra trading hour at night (95% CI: 9–24%). The estimates from the sensitivity analyses were also statistically significant; 22% per extra hour according to the pooled ARIMA estimates, and 13% per extra hour for the change model.

**Table 3:** Estimated effect of restaurant closing hours on assaults in city centre, including control for assaults in city periphery. Estimates based on (1) fixed-effects (FE) modelling, (2) pooled city-specific ARIMA modelling and (3) change model.

	*Estimate*	*SE*	*95% CI*	*P-value*
FE model*				
Hours	4.80	1.12	2.60, 6.99	<0.001
Control	0.20	0.07	0.07, 0.33	0.002
Pooled ARIMA				
Hours	6.31	1.74	2.90, 9.72	<0.001
Control	0.19	0.07	0.05, 0.33	0.007
Change model				
Hours	3.94	1.72	0.56, 7.31	0.028
Control	0.92	0.36	0.21, 1.63	0.010

* R^2^ = 0.450. SE: standard error; CI: confidence interval.

Turning to the issue of whether or not the influence of closing hours is symmetrical, our findings suggested that this is indeed the case; the estimated effects of both extended and restricted closing hours were statistically significant and of the same magnitude (Table 4). The estimated relative effects per hour were in the range 19–21% in the FE models and 22–24% in the ARIMA models. The estimates were not statistically significantly different; the *t*-tests equalled 0.25 and 0.14 for the difference between the FE estimates and the ARIMA estimates, respectively.

**Table 4:** Estimated effect of extended and restricted on-premise closing hours on assaults in city centre, including control for assaults in city periphery. Estimates based on (1) fixed effects (FE) modelling and (2) pooled city-specific autoregressive integrated moving average (ARIMA) modelling.

	*Extended closing hours*				*Restricted closing hours*			
		
	*Est*	*SE*	*95% CI*	*P-value*	*Est*	*SE*	*95% CI*	*P-value*
FE model								
Hours	6.22	1.43	3.42, 9.02	<0.001	5.53	2.56	0.51, 10.55	0.031
Control	0.12	0.20	−0.28, 0.51	0.554	0.27	0.08	0.11, 0.43	0.001
Pooled ARIMA								
Hours	7.03	2.00	3.11, 10.95	<0.001	6.54	2.92	0.82, 12.26	0.025
Control	0.16	0.20	−0.23, 0.55	0.424	0.26	0.09	0.08, 0.44	0.004

SE: standard error; CI: confidence interval.

## DISCUSSION

By analysing a series of natural experiments of changes in closing hours for on-premise alcohol sales in Norway, we found that even small changes (≤ 2 hours) appear to have an impact on night-time violence in inner-city areas. A 1-hour change in closing hours for on-premise sales was accompanied by an approximately 20% change in violent crime rates at weekend nights in city centres. These findings are in line with a few previous studies with rigorous research design [20,23]; i.e. Chikritzhs & Stockwell found that a 1-hour extension of closing hours was accompanied by a significant increase in night-time assaults in and around hotels with extended trading permits [20], and Kypri and co-workers [23] found that a 1.5–2-hours restriction in closing hours was associated with a significant decrease in night-time assaults in the city centre. In the latter study, the intervention comprised other measures as well, such as lock-out [23]. It should be noted that although the findings point in the same direction, the magnitude of the estimated impact is not comparable across these studies because the interventions and the outcome measures differ. Moreover, the present study adds to the literature by demonstrating symmetry in the impact of changes in closing hours on violence rates; i.e. a 1-hour extension of closing hours appears to have a similar impact on violent crime as a 1-hour restriction of closing hours.

### Strengths and limitations

The estimates of the impact of a change in closing hours on reported violence at weekend nights from three different analytical approaches were of the same magnitude, which suggests that the findings are quite robust. By collecting more detailed information on location, number and types of premises that were affected by changes in closing hours, we have probably obtained a more precise exposure measure and relevant control measure than is often the case in such studies. We also obtained data on other simultaneous changes that concern on-premise licences, but we had no indications that any of these were likely to have confounded the relationship. The pooling of estimates from many cities most probably countered the problem of low test power due to a modest number of assaults in each of the relatively small cities.

Nevertheless, the input series (closing hours) comprised some interpolations and thus imprecise measures, which is likely to imply that the parameter estimates may be somewhat deflated [35]. Data on violence from other sources (e.g. emergency rooms) would have been valuable to validate those from the police reports, but such data were not available. Other information that would have been of interest includes intoxication level among bar patrons and whether the assaults occurred inside the licensed premises. This could shed more light on the mechanisms underlying the observed association between closing hours and violence.

### Implications

Changes in closing hours may be considered politically feasible, as they are easily implemented and sustained and imply no direct economic costs to the authorities. On these grounds, the findings of this study suggest that even minor restrictions in closing hours for on-premise alcohol sales could be an attractive measure to curb night-time assaults in inner city areas. The findings also provide evidence-based arguments against the relaxation of the trading hours that is commonly promoted by the industry. Other possible successful strategies to prevent violence in the night-time economy, such as the ‘STAD-project’ in Stockholm [36] and ‘Safer bars program’ in Toronto [37] may be viewed as attractive supplements to restricted closing hours, possibly reinforcing the effects of each other.

Policy makers and other actors in the alcohol policy arena have often applied simple comparisons of data before and after a policy change. Corresponding to the various findings reported from the studies of the 2003 Licensing Act [16–18], Norwegian media and policy makers have also reported conflicting findings with respect to changes in violence rates following changes in on-premise closing hours in Norwegian cities. The findings of this study therefore illustrate the importance of not drawing conclusions from an intervention in one small city and of applying a strong design and reliable measures when evaluating an intervention. Future research may benefit from supplementary studies that may shed more light on underlying mechanisms.

## Declarations of interest

The study was funded by Norwegian Institute for Alcohol and Drug Research and the Swedish Council for Working Life and Social Research. The authors have no connections with the tobacco, alcohol, pharmaceutical or gaming industries or any body substantially funded by one of these organizations.
